# Pronounced impairment of B cell differentiation during bone regeneration in adult immune experienced mice

**DOI:** 10.3389/fimmu.2025.1511902

**Published:** 2025-03-03

**Authors:** Mireille Ngokingha Tchouto, Christian H. Bucher, Ann-Kathrin Mess, Simon Haas, Katharina Schmidt-Bleek, Georg N. Duda, Dieter Beule, Miha Milek

**Affiliations:** ^1^ Julius Wolff Institute of Biomechanics and Musculoskeletal Regeneration, Berlin Institute of Health at Charité – Universitätsmedizin Berlin, Berlin, Germany; ^2^ Core Unit Bioinformatics, Berlin Institute of Health at Charité–Universitätsmedizin Berlin, Berlin, Germany; ^3^ Berlin-Brandenburg School for Regenerative Therapies, Charité – Universitätsmedizin Berlin, Freie Universität Berlin and Humboldt-Universität zu Berlin, Berlin, Germany; ^4^ Berlin Institute of Health (BIH) Center for Regenerative Therapies, Berlin Institute of Health at Charité - Universitätsmedizin Berlin, Berlin, Germany; ^5^ Systems Hematology, Stem Cells & Precision Medicine, Berlin Institute of Health at Charité-Universitätsmedizin Berlin, Berlin, Germany; ^6^ Berlin Institute for Medical Systems Biology, Max Delbrück Center for Molecular Medicine in the Helmholtz Association, Berlin, Germany; ^7^ Max Delbrück Center for Molecular Medicine in the Helmholtz Association (MDC), Berlin, Germany

**Keywords:** immune experience, bone healing, osteotomy, B cell, differentiation, adaptive immunity

## Abstract

**Introduction:**

Alterations of the adaptive immune system have been shown to impact bone healing and may result in impaired healing in some patients. Apart from T cells, B cells are the key drivers of adaptive immunity. Therefore, their role in age-associated impairments of bone healing might be essential to understand delays during the healing process. B cells are essential for bone formation, and their dysfunction has been associated with aging or autoimmune diseases. But whether age-associated changes in B cell phenotypes are involved in bone regeneration is unknown.

**Methods:**

Here, we aimed to characterize the role of immune aging in B cell phenotypes during the early inflammatory phase of bone healing. By comparing non-immune experienced with young and immune experienced mice we aimed to analyze the effect of gained immune experience on B cells. Our single cell proteo-genomics analysis quantified thousands of transcriptomes of cells that were isolated from post osteotomy hematoma and the proximal and distal bone marrow cavities, and enabled us to evaluate cell proportion, differential gene expression and cell trajectories.

**Results:**

While the B cell proportion in young and non-immune experienced animals did not significantly change from 2 to 5 days post osteotomy in the hematoma, we found a significant decrease of the B cell proportion in the immune experienced mice, which was accompanied by the decreased expression of B cell specific genes, suggesting a specific response in immune experienced animals. Furthermore, we detected the most extensive B cell differentiation block in immune-experienced mice compared to non-immune experienced and young animals, predominantly in the transition from immature to mature B cells.

**Discussion:**

Our results suggest that the pronounced impairment of B cell production found in immune experienced animals plays an important role in the initial phase leading to delayed bone healing. Therefore, novel therapeutic approaches may be able target the B cell differentiation defect to retain B cell functionality even in the immune experienced setting, which is prone to delayed healing.

## Introduction

Bone healing following an injury or surgery is a physiological process that in almost all cases leads to full reconstitution and tissue regeneration (1, 2). Almost all individuals will at some point in their life undergo such a cascade of full bone regeneration but in an estimated number of 10-20% of cases, healing is delayed or absent (1, 3). Successful healing requires an orchestrated sequence of cellular and molecular events that initially include a pro- and anti-inflammatory phase (1). After fracture, a hematoma is formed from coagulating blood, bone debris and invading cells from its surrounding tissue (4). Cells that function in the innate and adaptive immune response contribute to the bone healing process. Apart from macrophages, neutrophils and cytotoxic T lymphocytes contribute to the pro-inflammatory phase by releasing cytokines to initiate an inflammatory state (4). In elderly patients, this phase can be prolonged, which may result in delayed or impaired bone healing (5–8).

Previous work has shown that delayed healing is associated with advanced immune experience (9). Furthermore, in elderly patients, cells that characterize an increased adaptive immune response have been shown to impact bone healing (10). Several cellular phenotypes have been identified that alter the healing outcome in the elderly, eventually resulting in delayed healing (11, 12). In aged individuals, the differentiation of hematopoietic stem cells (HSC) towards myeloid cells was found to be promoted and resulted in lower numbers of B and T lymphocytes (13–15). In addition, in humans the proportion of naive T cells decreased during aging (16, 17), whereas the proportion of memory T cells increased (18–20), and was correlated with prolonged healing (2, 9).

However, B cells and their phenotypes – also a key element of adaptive immunity – have surprisingly been less researched, even though they may be highly important during the inflammatory phase of bone healing (21, 22). In addition, B cell dysfunction has been associated with aging or autoimmune diseases of the bone (23–25). Atypical B cells are formed in B cell compartments, and harbor distinct phenotypes such as altered gene expression profiles, proliferation, and variation in B cell receptor repertoires (23). Since dysfunctions of B cells are common in the aging population, alterations of B cell phenotypes might be prominent determinants of impaired, delayed or pro-longed bone healing by affecting the pro-inflammatory phase and/or later stages post fracture.

Investigation of the role of B cells in bone healing in young mice by Könnecke et al. (26) and Zhang et al. (27) revealed that the proportion of B cells decreased during the inflammatory phase but was restored 14 days after fracture. In addition, immunomodulatory properties of B cells have been recently investigated in the context of bone healing in human patients (21, 22). The results of these studies suggest that specific B cell functions are required for favorable bone healing outcome but provide little insight into the effect of altered B cell phenotypes on bone healing in elderly individuals. It remains unclear what effect chronological aging has on the B cell phenotype and how immune-aging affects B cells and their function during bone healing (22).

Here, we investigated the cellular and molecular characteristics of B cells during early bone healing in a mouse osteotomy model (28). We aimed at differentiating the effect of chronological aging from gained immune experience at the early time points during bone healing and therefore compared three female mouse groups which corresponded to differential bone healing outcomes. We observed the strongest alteration of the B cell phenotype in the hematoma of immune experienced mice from 2 to 5 days post osteotomy, particularly in the impairment of immature to mature B cell differentiation, which was accompanied by the decreased expression of several B cell specific genes, and could be a result of increased expression of interferon associated genes in monocytes and decreased expression of interferon receptors in B cells. While non-immune experienced animals showed a smaller B cell differentiation block, the most pronounced impairment was detected in immune experienced animals. Our work therefore suggests that the altered B cell phenotype may contribute to delayed healing in elderly patients.

## Materials and methods

### Animal experiments

**Figure 1:**
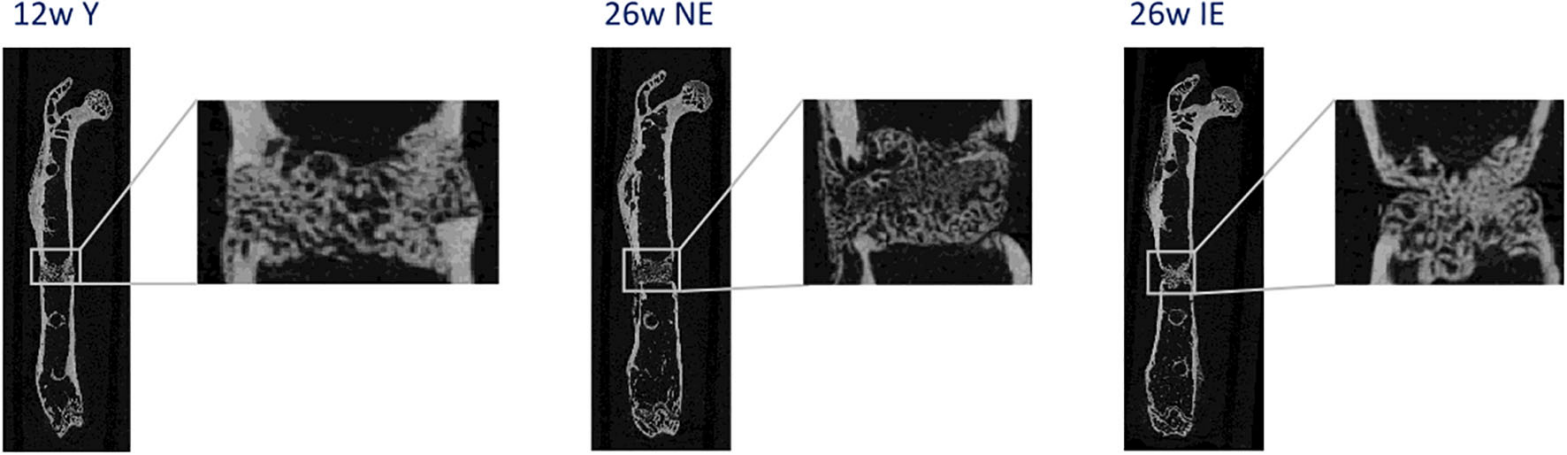
Representative radiographs of the healing process 21 days after osteotomy for young (left), NE (middle) and IE (right) animals stabilized with an external fixation device.

C57BL/6N female mice were purchased from Charles River Laboratories at an age of 6 weeks. Different mouse age at the time of osteotomy and different housing conditions enabled the comparison of 3 types of mice: 12 weeks old, maintained under non-specific pathogen free (SPF) conditions, 26 weeks old under SPF conditions and 26 weeks old under non-SPF conditions. For this purpose, mice were housed under SPF conditions (non-experienced immune status, NE), meaning that individually ventilated cages were placed behind a barrier (change of clothing, masks and gloves), while non-SPF mice (experienced immune status, IE) were housed in cages with grid lid in a room that was freely accessible. All mice were monitored according to FELASA guidelines and were healthy. Non-SPF mice encountered environmental pathogens inducing an immunological memory, changing their naïve T cell pool slowly and individually towards higher percentages of effector memory T cells. This experimental design has been published previously for 3 and 12 months old animals (10). To confirm that the differences between IE and NE animals that we had observed in 3 and 12-months-old mice could also be confirmed in the 6-months-old mice, radiographics of the femurs were taken at 21 days after osteotomy (exemplary images displayed in **Figure 1**). In the young immune-naïve animals (**Figure 1**. left; young), complete bridging of the osteotomy gap was seen neatly aligned with the cortical bone and bone marrow cavity already re-opened, indicating callus remodeling. In the chronologically older animals (**Figure 1** middle; NE) bridging of callus was only seen on one side while not yet bridged on the cisco cortex with no signs of remodeling of the marrow cavity present. In the age-matched immune experienced group (**Figure 1**, right; IE) the callus was not filling the osteotomy gap, the cortical bone ends were not bridged, showed thinner ends with signs of bone resorption. This resembled the capping of the marrow channel seen in delayed or non-union cases. The female mouse models used are illustrated by the representative images, each which match well our earlier described female mouse model data for 3 and 12 months old animals (PMID: 36028760). Overall, female mice show a retarded healing compared to their male counterparts and thus represent a more valid animal model system in regard to bone healing in human (PMID: 34434120, 20652815).

Bone regeneration was studied using the above-mentioned 3 mouse types following osteotomy of the left femur and fixation such that fast and effective healing would be enabled (external fixation by a rigid MouseExFix, RISystem, Davos, Switzerland). Briefly, mice were anesthetized with a mixture of isoflurane and oxygen. After initial shaving of the thigh the fixation was positioned with two proximal and distal pins. Thereafter, a 0.7 mm osteotomy was introduced between the middle pins by using a Gigli wire (RISystem, Davos, Switzerland). The first line analgesia was done with Bubrenorphine pre surgery, antibiotics with clindamycine. Tramadol was added post-surgery to the drinking water for 3 days.

### Mouse osteotomy

Animals were intraperitoneally injected with a mixture of medetomidine and ketamine to induce deep anesthesia. Thereafter, animals were euthanized by cervical dislocation. The osteotomized femur was extracted and stored for transportation in ice cold phosphate-buffered saline (PBS). The fracture gap and proximal and distal regions from the osteotomy were separated and single cell suspensions were taken by flushing the bones and filtering the probes through cell culture meshes for each of these regions separately. For sorting, cells were stained with fluorescently labeled antibodies, oligonucleotide labeled hashtag antibodies and oligonucleotide labeled surface protein antibodies (TotalSeq, BioLegend, San Diego, USA) before pooling the sample for three animals each. An enrichment sort for low abundant cells was performed by decreasing highly abundant cells (staining: Ly6G, CD45R/B220, CD11c, Live/Dead, Ter119, CD41) before cell encapsulation according the 10X Genomics protocol. With 2 timepoints post osteotomy (5 and 2 days) and 3 regions for each bone (hematoma, proximal and distal), this experimental setup consisted of 18 experimental groups with 3 mice per group, i.e. 54 mice in total.

### Library preparation and high-throughput sequencing

Chromium Next GEM Single Cell 3’ Reagent Kits v3.1 (Dual Index) with Feature Barcode technology for Cell Surface Protein (CG000317 Rev C) was used according to the manufacturer’s protocol to construct the libraries. Libraries were sequenced on the Illumina NovaSeq 6000 sequencer. Demultiplexing was carried out with bcl2fastq (v2.20, Illumina) with default parameters.

### Single cell RNA-seq data processing

Cellranger count v7.1.0 (10X Genomics) was used to process the raw sequencing reads, which included alignment to the mouse genome (mm10) and generation of count matrices with mRNA and ADT read counts. The count data was then processed according to standard Seurat (29) (v4.1.1) processing. Demultiplexing was carried out within Seurat using the HTODemux function. This allowed us to remove doublets. In total, 14810 (58.55%) of all droplets were single cells with a median of 2100 expressed mRNAs. Next, principal component analysis (PCA) was carried out by using regression of the difference between the G2M and S cell cycle gene scores. We completed the pre-processing step of the RNA assay by filtering cells based on the number of mRNAs per cell (cells with >6500 mRNAs/genes were removed), the percentages of mitochondrial (cells with <10% were removed) and ribosomal (cells with <25%) genes. This resulted in the recovery of 87.19% (n = 12913) cells. In addition, the protein expression (ADT) data was normalized using Seurat’s centered log ratio method. Moreover, the individual samples were integrated using FindAnchors and IntegrateData functions to minimize potential batch effects. We investigated the effect of different covariates (sample, timepoint, batch, group) on the results of the data integration. We found that data integration, where we compensated for group-based batch effects, gave the most reproducible result in terms of cell cluster composition. We re-ran the PCA on the significantly variable genes, and the top 20 principal components were selected as input for both UMAP embedding and clustering. For clustering, we used the FindClusters function implemented in the Seurat package, specifying the resolution of 1.0.

### Cell annotation

Seurat’s FindAllMarkers function was used to identify the differentially expressed genes in each cluster compared with all other clusters. To annotate the clusters, we combined Seurat’s MapQuery function using the Tabula Muris (30) droplet dataset as a reference. In addition, we used manual annotation by applying key marker genes for specific cell types that were based on previously published studies (31, 32). Once the cell types were annotated, we calculated the cell type proportions in each sample.

### Differential expression analysis

Due to a low number of cells detected, we summed the gene-wise read count for each sample over all cells and performed differential pseudobulk expression analysis with DESeq2 (33). The proximal, hematoma and distal bone marrow samples from one animal were considered as paired samples. This was considered in the DESeq2 model design by including the mice ID information (i.e. ~mouseID + group). Pairwise comparisons between different groups were extracted from the same results object, such that total variance from all samples in the experiment was considered.

To perform gene set enrichment analysis on the DESeq2 results, we used tmod (34), whereby a gene list sorted by DESeq2 P value was input into tmodCERNOtest function. As a threshold for tmod results, which outputs adjusted P value, as well as effect size (AUC), we considered enriched pathways to have an AUC>=0.65, and an adjusted P value < 0.05. To define genes with increased or decreased expression we used a cutoff of adjusted P value <0.05 and absolute log2-transformed fold change of >0.5. The immune related gene sets for pathway enrichment, were provided by the tmod package and are publicly available (35, 36).

We used tmod’s ggEvidencePlot function to visualize the genes in specific B cell pathways. We then used this list of genes as input to the Seurat AddModuleScore function to estimate the cell-wise score for this gene set and to determine, in which cell types they were mainly expressed. To exclude potential effects of different sized groups for comparison, we randomly downsampled our results to 500 cells for each group. We then visualized the difference between the IE hematoma at 2 days and 5 days with a boxplot and performed a Wilcoxon rank sum test to assess significance at *p < 0.05, **p < 0.01 and ****p< 0.0001.

### Pseudotime analysis

For trajectory inference, we used Slingshot (37) and Monocle3 (38). This analysis was carried out only on B cells (pre/pro-B, immature and mature) and the hematopoietic precursor cells (HPCs), which were sub-clustered using the Seurat FindClusters function. Due to the low number of plasma cells detected per sample and because of vast differences in their transcriptional profile in comparison with other B cells, we did not include them in the trajectory analysis. After clustering, we manually re-annotated the new clusters of HPCs and B cells using marker gene expression information. Next, we converted the Seurat object into the SingleCellExperiment (39) (sce) object and provided it as input to Slingshot. For the pseudotime trajectory estimation, we used HPCs as the starting cluster.

Wilcoxon rank sum test was used to compare cell-wise pseudotime values between mice groups with significance at *p < 0.05, **p < 0.01 and ****p< 0.0001. For trajectory inference analysis using Monocle3, we provided the same Seurat object as for Slingshot. The data were then transformed into a monocle main class “cell_data_set” and, using the learn_graph function, we were able to fit a main graph to each partition. The cells were then ordered according to pseudotime by manually selecting the nodes in the graph that indicate the start of the trajectory. Here, we selected the nodes corresponding to the hematopoietic precursor cells as our roots.

## Results

### Single cell analysis of murine B cells post osteotomy

To better understand the role of B cells in bone healing, we assayed immune cells in the bone marrow (BM) osteotomy microenvironment at the single cell level. Since bone healing outcome is known to be negatively affected by ageing, we used a previously published mouse model (28) to compare 12 and 26 weeks old mice. In addition, to differentiate the effect of chronological ageing from gained immune experience, we compared female animals maintained either in the specific pathogen free (SPF) environment (non-immune experienced, NE), or the non-SPF environment (immune experienced, IE). Due to known differences in the healing progression within different regions of the fracture microenvironment, we studied differences in their immune cell composition by dissecting 3 different anatomical regions from each bone (hematoma, proximal and distal). To gain insight into the early cellular dynamics during the inflammatory phase of bone regeneration, mice were sacrificed 2 and 5 days post osteotomy. An overview of the experimental groups, which were defined by mouse type, compartment, and time points post osteotomy are given in **Supplementary Table S1**.

**Figure 2:**
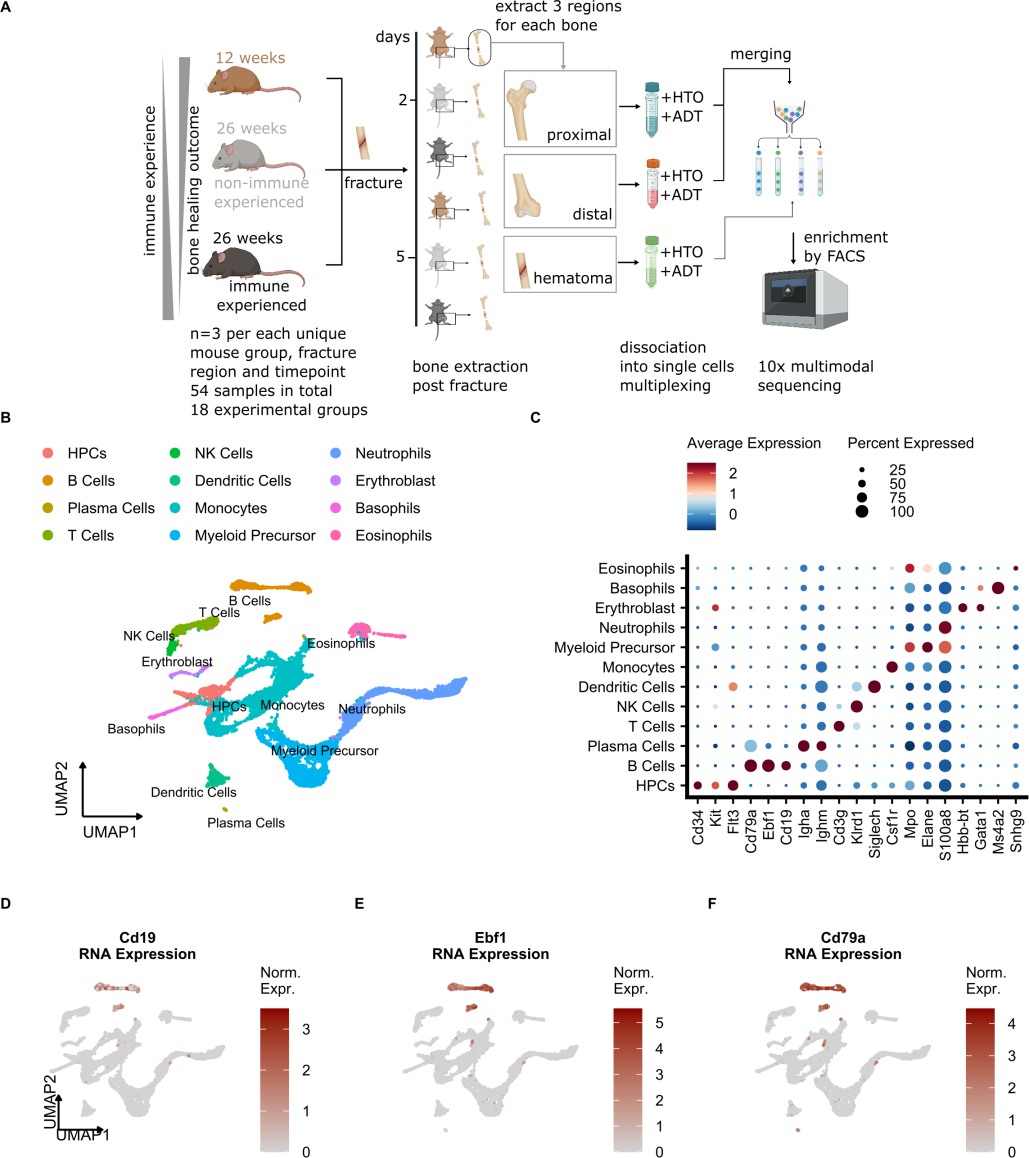
Single cell analysis of the mouse osteotomy microenvironment detects common immune cell types. **(A)** Schematic of the experimental setup. 12-week (young), 26-week specific-pathogen-free (SPF, non-immune experienced, NE) and 26-week non-SPF (immune experienced,IE) mice undergoing osteotomy (n=3 per group). After 2 and 5 days, the mice were sacrificed and the proximal, hematoma and distal bone marrow were harvested from each bone. After washing of the bone sections, the individual cells were dissociated, and labeling of cells with oligonucleotide-tagged antibodies for cell hashing (HTO) and detection of other cell-surface proteins (ADT, Cd19, Cd4, Cd8a, Cd3, Cd335) was performed. After pooling, cells were sorted by a multi-layer FACS approach, followed by single-cell droplet encapsulation with microbeads, library preparation and next-generation sequencing. **(B)** UMAP embedding of cell types detected in this study. Annotated cell clusters are shown in different colors. **(C)** Dot plot of average normalized mRNA expression values and percentage of all cells that express a given cell type marker. **(D-F)** UMAP embedding of cells detected in this study showing the cell-wise normalized expression of B cell marker genes.

To overcome common technical biases, the single cell experiment was carried out using hash-tag multiplexing (**Figure 2A**). After cell dissociation, enrichment for white blood cells, labeling, cell encapsulation and library preparation were performed, followed by the multi-modal single cell assay, which included transcriptome quantification and surface protein detection (**Figure 2A**) (40). We achieved high recovery of cells after the demultiplexing procedure, which in addition allowed us to bioinformatically remove doublets (**Supplementary Figure S1C**). After quality control filtering, standard single cell data processing (see Methods for details) led to the quantification of 12913 single cell transcriptomes. Clearly separated cell clusters (**Supplementary Figure S1A**) were obtained and showed expression of specific gene expression markers (**Supplementary Figure S1B**). A combination of manual annotation and projection of query cells onto a murine BM reference (30) (**Supplementary Figure S1D**) resulted in the identification of 12 cell types (**Figure 2B**), including expected major immune cell types such as monocytes, neutrophils, T and B cells. Expected mRNAs were expressed in the detected cell types (**Figure 2C**), such as Cd3g for T cells, Cd79a, Ebf1 and Cd19 for B cells, as well as Cd34, Kit and Flt3 for hematopoietic precursor cells (HPCs).

To evaluate total bone marrow composition, we next calculated cell type proportions in NE and IE mice and compared them with other published bone marrow datasets (10, 27, 30, 31). We found that the highest cell proportions in our dataset corresponded to granulocytes and monocytes, followed by B and T cells, which was confirmed by the previously published results (**Supplementary Figure S1E**). According to the mRNA expression of B cell marker genes (**Figures 2D-F**, **Supplementary Figure S1B**), we were able to detect 3 clusters (13, 15 and 27), which according to the marker gene expression (Fcer2a, Cd38, Vpreb1) corresponded to the mature, immature and pre/proB differentiation states, respectively (**Supplementary Figure S1B**). Our analysis also detected a low number of plasma cells, which are commonly underrepresented in BM scRNA-seq (41) and not located in neighboring clusters with respect to other B cell subtypes (42) because their transcriptional profiles are vastly different. Therefore, we excluded plasma cells from our downstream B cell proportion and trajectory inference analysis (see Methods for details). In summary, the major BM cell types including B cell subtypes were successfully detected in our model system.

### B cell proportion decrease in the hematoma of IE but not in NE and young mice

**Figure 3:**
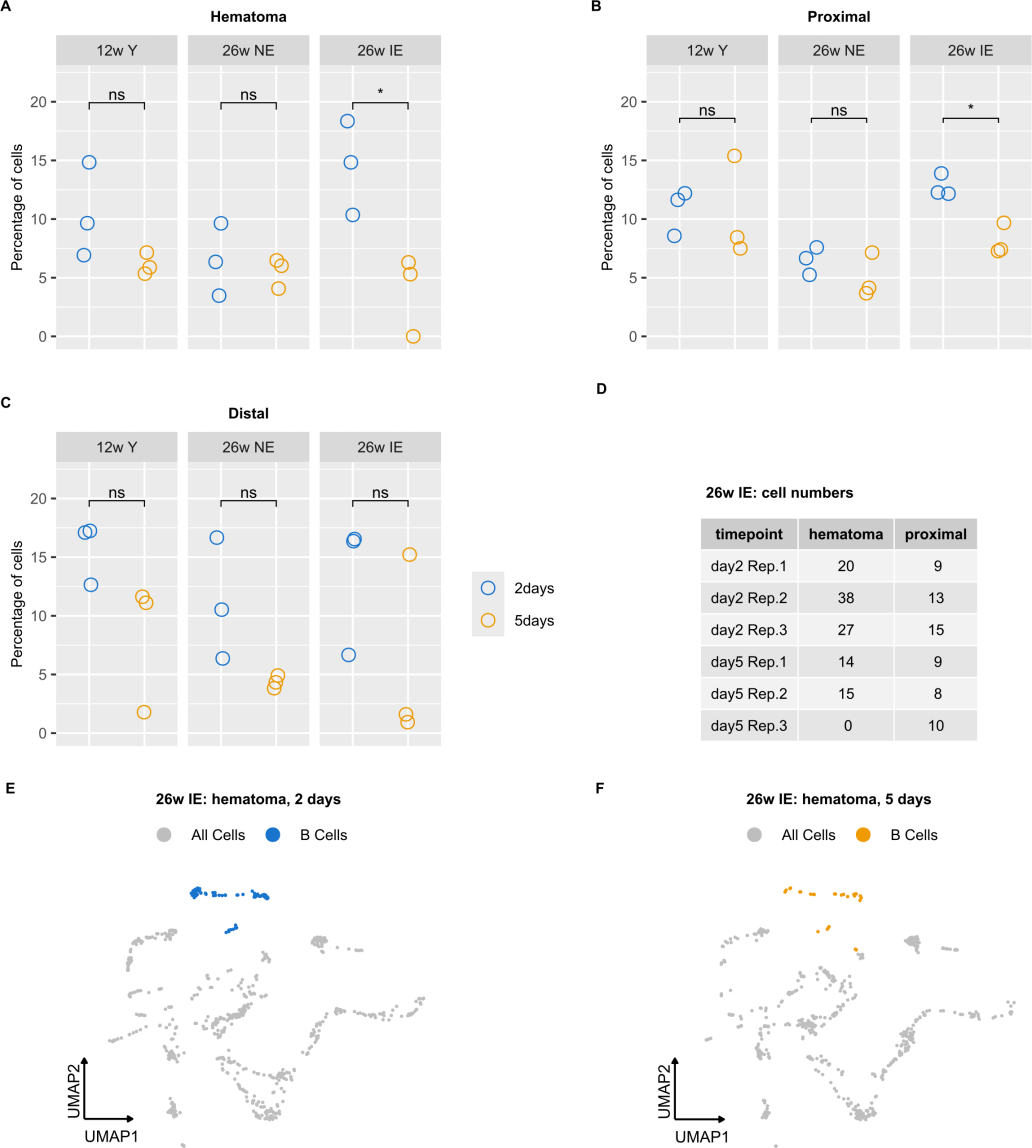
B cell proportion analysis in different mouse groups reveals a decrease in B cells detected in IE animals. **(A-C)** Percentages of B cells were calculated for each sample, i.e. 2 (blue) or 5 days post osteotomy (orange) with respect to the different bone regions (hematoma, proximal and distal) and the different mouse groups (young, NE, IE). Significance was evaluated using unpaired two-sided Student’s t-test and differences are indicated with an asterisk (*p < 0.05). **(D)** Absolute B cell number in the hematoma of IE mice for each animal on days 2 and 5. **(E, F)** UMAP embedding of cells detected in the hematoma of IE mice, with B cells highlighted in blue (day 2) and orange (day 5).

We next evaluated the differences in B cell proportion between the mouse groups. We thus calculated cell proportions as percentage of total cells detected in each sample (**Supplementary Table S2**). No differences in B cell proportions were observed between different compartments (hematoma vs. proximal vs. distal) 5 days post osteotomy (**Supplementary Figures S2A, B**). However, when comparing B cell proportions from 2 to 5 days post osteotomy in the same mouse type and within the same compartment, we observed a significant decrease in the proportion of B cells in the hematoma of IE mice (**Figure 3A**). In addition, a less extensive decrease occurred proximal to the osteotomy (**Figure 3B**). We did not observe any significant change in the B cells proportion in the area distal to the osteotomy (**Figure 3C**). The decrease in B cell proportion in the hematoma and proximal bone marrow was only detected in the IE mice but not in the NE or young mice. We also inspected the absolute number of B cells in these groups of mice, which showed consistently lower absolute numbers 5 days post osteotomy in the hematoma but not in the tissue proximal to the osteotomy (**Figure 3D**). The absolute B cell decrease was also apparent from the low-dimensional visualization of cells in the hematoma of IE animals 5 days post osteotomy (**Figures 3E, F**).

In summary, the specific decrease in B cell proportion detected in the IE animals could result both from the lower absolute number of B cells, or from an increase in cell numbers of other cell types. These results indicated either the impairment of B cell maturation and/or increased B cell death specific for the hematoma and, to a smaller extent in the proximal tissue of IE mice.

### Decreased expression of B cell specific genes in the hematoma of IE animals

**Figure 4:**
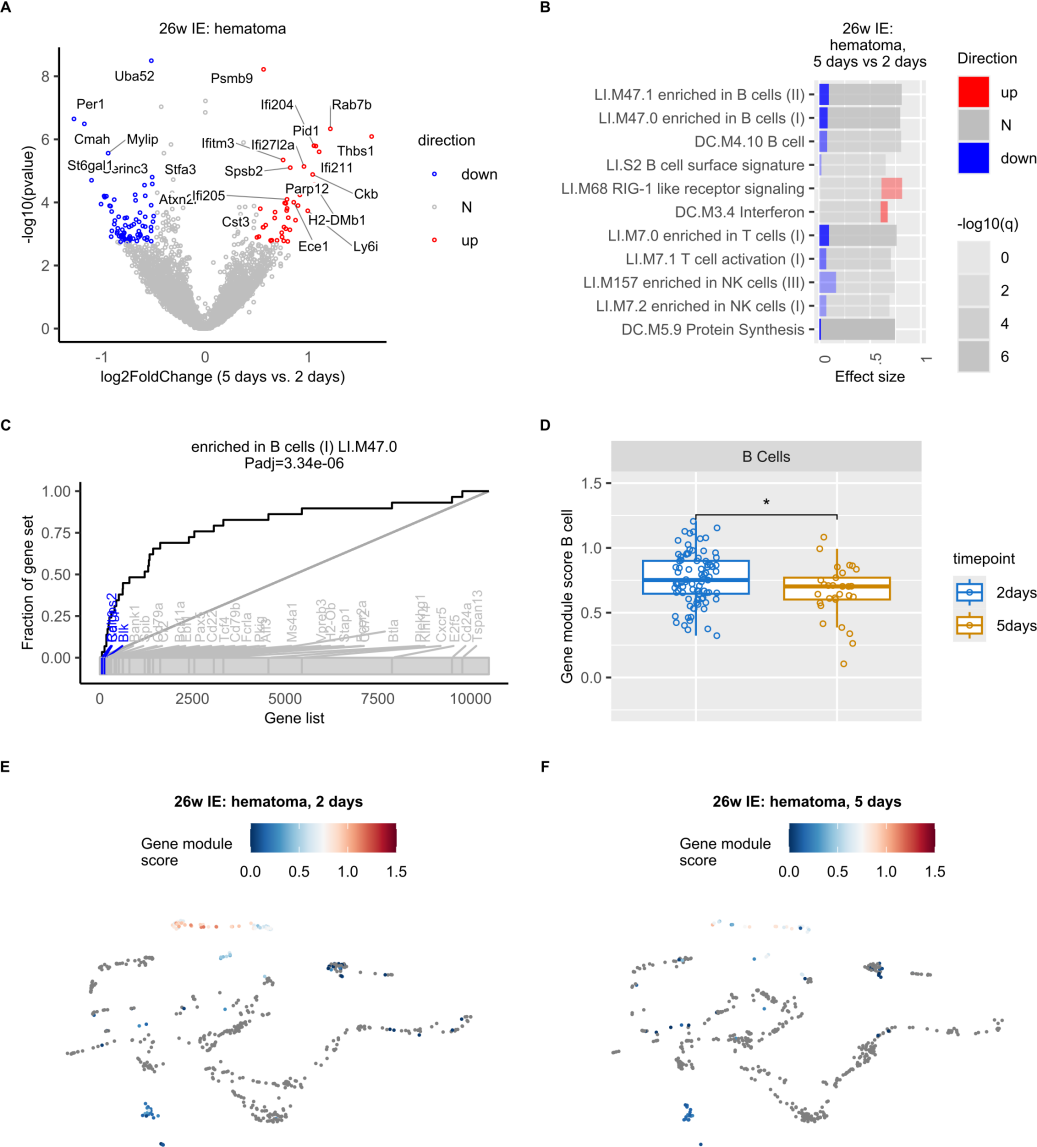
Decreased expression of B cell specific genes in the hematoma of IE mice 5 days post osteotomy. **(A)** Volcano plot of log2-transformed fold changes (5 days vs. 2 days in the hematoma of IE mice) versus negative log10-transformed p-values (Wald test). Differentially expressed genes (adjusted p-value < 0.1 and absolute log2-transformed fold change > 0.5) are marked in red (up, increased), blue (down, decreased), or grey (N, non-differential). **(B)** Panel plot of gene set enrichment analysis of differentially expressed genes shown in a). Bars represent relative fractions of mRNAs with decreased (blue) expression, not-differential (N) genes and increased (red) expression within each of the gene sets. **(C)** Evidence plot depicting area under the curve (AUC) as a measure of effect size for the gene set term “enriched in B cells (LI.M47.0) “. The ranking of genes in the gene set within all considered genes is denoted with the bottom grey ribbon. **(D)** Box plots of LI.M47.0 gene module scores per cell at 2 and 5 days post osteotomy in the hematoma of IE mice. Wilcoxon rank sum test was used for comparisons. The lower and upper hinges of box plots correspond to the 25th and 75th percentiles, respectively. Center lines of box plots depict the median values. Significant differences are indicated with asterisks (*p< 0.05). **(E, F)** UMAP embedding showing the gene set (LI.M47.0) scores in IE mice 2 **(E)** or 5 days post osteotomy **(F)**.

Next, we aimed to define the underlying molecular characteristics of the decrease in B cell proportion detected in the hematoma of IE mice. Due to the low number of detected cells per sample, we were not able to carry out differential expression analysis on B cells only. Instead, our pseudobulk differential expression analysis (see Methods) in hematoma from 2 days to 5 days post osteotomy revealed altered expression of several key immune-related mRNAs in IE mice (**Figure 4A**). Compared to the hematoma of young and NE animals (**Supplementary Figures S3A, B**) the IE animals showed higher absolute fold changes from 2 to 5 days post osteotomy, suggesting most extensive changes in gene expression in IE animals.

To achieve a better understanding of the function of genes with differential expression 5 days post osteotomy, we performed gene set enrichment analysis. We found a significant enrichment of gene sets such as “enriched in B cells” and “B cell”. Interestingly, the differentially expressed genes in the gene set “enriched in B cells” showed exclusively decreased expression 5 days post osteotomy (**Figure 4B**). And included well-known B cell specific genes (**Figure 4C**), such as Ralgps2, Cd19, Cd79a, Ebf1, Pax5, Vpreb3, Cd24 (32), as well as Blk, which was previously found to be differentially expressed in mature B cells (32, 43). We did not identify any significantly enriched gene sets in young and NE animals.

To exclude that the results of our pseudobulk analysis was a consequence of the decreased expression of B cell specific genes in cell types other than B cells, and/or decreased number of B cells in IE mice, rather than a specific change in B cell gene expression program, we next computed cell-wise gene module scores for the “enriched in B cells”gene set identified in our pseudobulk pathway analysis. We observed the highest gene module scores in B cells (**Figures 4E, F**, **Supplementary Figure S3C**), confirming that the expression of B cell specific genes was the highest in B cells and not in other cell types. Furthermore, we found a significant decrease in gene module score 5 days post osteotomy (**Figure 4D**) and concluded that the expression of B cell specific genes was significantly decreased during bone healing of the post osteotomy hematoma in IE mice.

In summary, these results show a specific alteration of the B cell gene expression program in the hematoma of IE animals, where the most extensive decrease in B cell proportion also occurred.

### Impairment of hematoma B cell differentiation is most pronounced in IE mice and may be a result of altered gene expression programs in monocytes

**Figure 5:**
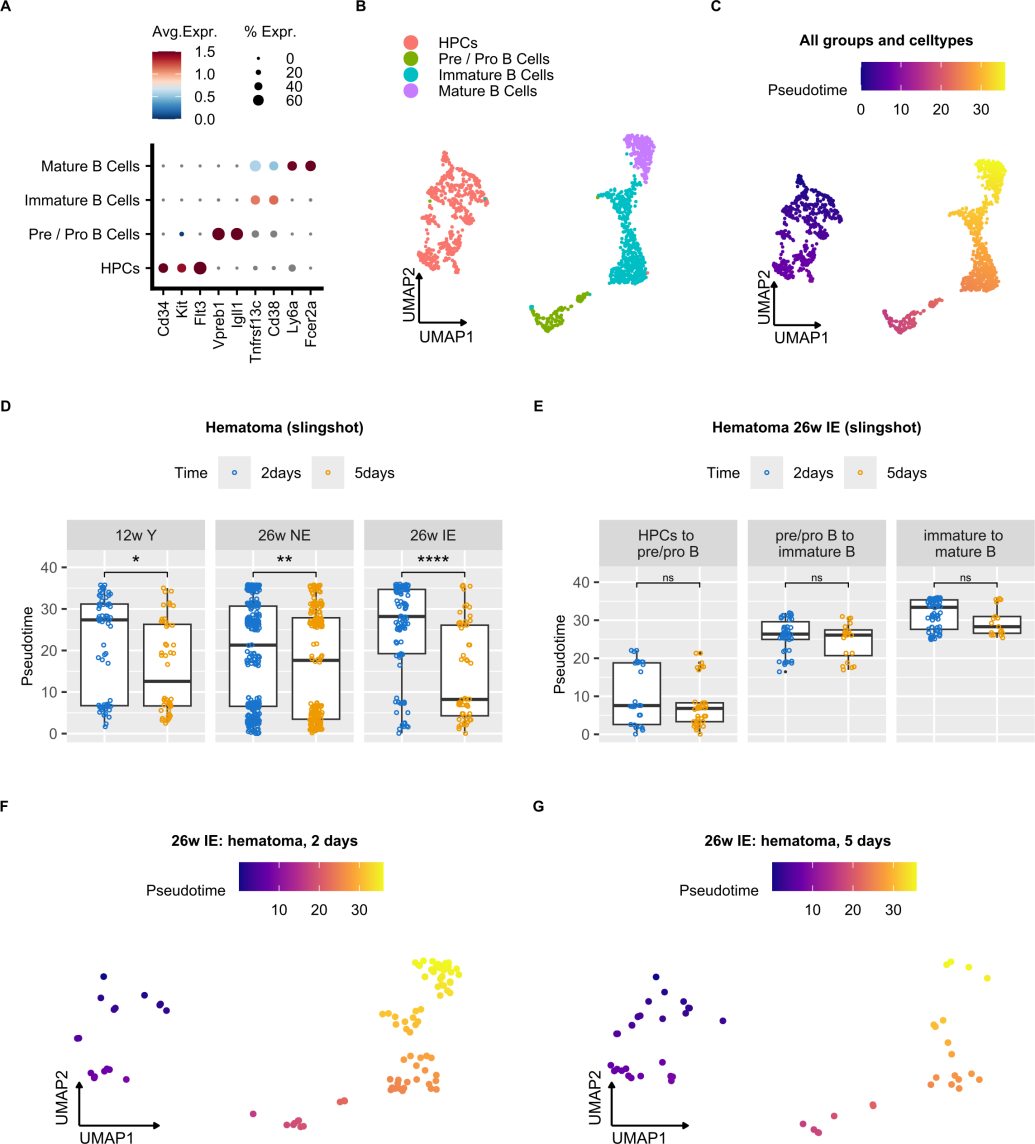
Impairment of B cell differentiation in the osteotomy hematoma of IE mice. **(A)** Dot plot showing average normalized mRNA expression values for the individual B cell subtypes and hematopoietic precursor cells (HPCs). **(B)** UMAP embedding of B cells and HPCs from all samples after sub-clustering. Annotated cell clusters are depicted in different colors. **(C)** UMAP embedding of B cells and HPCs with corresponding cell-wise pseudotime values. **(D)** Box plots of pseudotime values at 2 and 5 days post osteotomy in the hematoma of the different mouse groups (young, NE, IE) along the pseudotime trajectory. Wilcoxon rank sum test was used for comparisons. The lower and upper hinges of box plots correspond to the 25th and 75th percentiles, respectively. Center lines of box plots depict the median values. Significant differences are indicated with asterisks (*p < 0.05, ****p< 0.0001). **(E)** Box plots of pseudotime values 2 and 5 days post osteotomy in the hematoma of IE mice for different cell types along the pseudotime trajectory. Wilcoxon rank sum test was used for comparisons. **(F, G)** UMAP embedding of B cells and HPCs from IE mice 2 **(F)** or 5 days post osteotomy **(G)** with indicated cell-wise pseudotime values. ns, non significant; **p<0.01.

To compare the dynamics of B cell maturation during bone healing in different mouse groups, we applied the widely used trajectory inference approach termed Slingshot (37) (see Methods) on the B cell subtypes and HPCs (**Figure 5A**). After sub-clustering of these cells, which resulted in their clear separation (**Figure 5B**), we computed cell-wise pseudotime values that reflected the extent of cell differentiation. Expected pseudotime values for the examined cell populations that corresponded to their differentiation status were found, with HPCs showing the lowest values, followed by pre/pro, immature and mature B cells (**Figure 5C**).

To characterize the differential B cell dynamics in the post-osteotomy bone regions, we compared the cell-wise pseudotime values between different mouse groups (**Figure 5D**, **Supplementary Figure S4A, B**). We detected a significant decrease in pseudotime 5 days post osteotomy in the hematoma of all mouse groups (**Figure 5D**), indicating a general B cell differentiation block during the early inflammatory phase of bone healing. Nevertheless, the difference in the median pseudotime from 2 to 5 days post osteotomy was the highest in IE, followed by NE and young animals, suggesting that the impairment of B cell differentiation was most prominent in the IE background. Stratification of B cell subtypes that reflect differentiation states (**Figure 5E**) revealed a trend, which showed that the immature to mature B cell transition showed the most extensive pseudotime decrease from 2 to 5 days post osteotomy, suggesting that the transition from immature to mature B cells was most affected. In addition, we detected a higher number of de-differentiated cells with shorter pseudotime in IE mice of 5 days (**Figure 5G**) compared to 2 days post osteotomy (**Figure 5F**). To confirm these results, we also computed pseudotime values using an alternative trajectory inference method (Monocle 3) (44), recapitulating the Slingshot results (**Supplementary Figures S4C-E**). In summary, these results suggest that the impairment of hematoma B cell differentiation is most pronounced in IE mice and likely occurs during the transition from immature to mature B cells.

**Figure 6:**
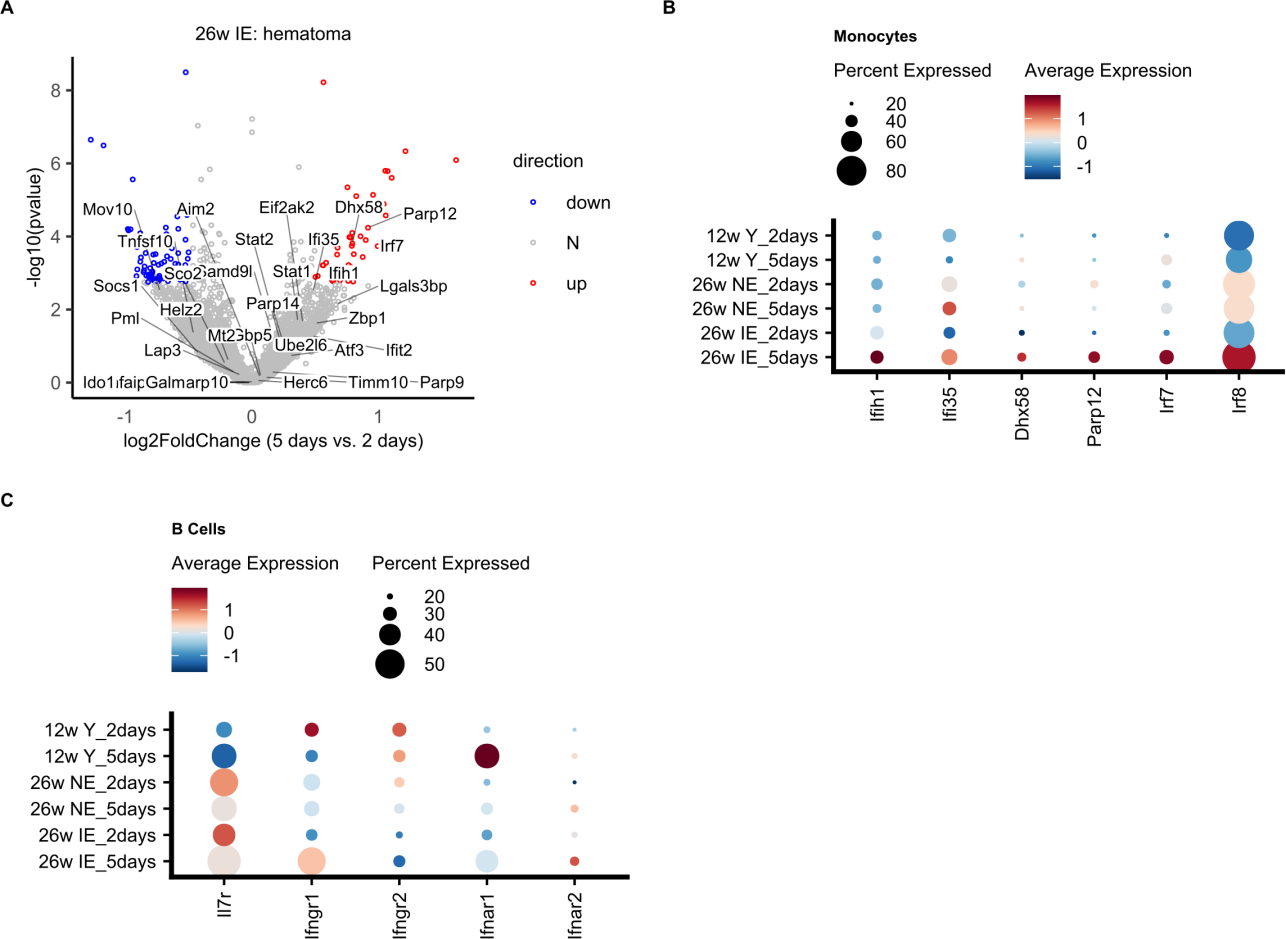
Differential expression of Ifn associated genes in monocytes and their target molecules in B cells. **(A)** Volcano plot of log2-transformed fold changes (5 days vs. 2 days of IE hematoma) versus negative log10-transformed p-values (Wald test). Genes included in the “Interferon” gene set (DC.M3.4) are indicated along with all differentially expressed genes (adjusted p-value < 0.1 and absolute log2-transformed fold change > 0.5), and colored in red (up, increased), blue (down, decreased), or grey (N, non-differential). **(B)** Dot plot showing the average normalized mRNA expression values and percentage of cells expressing a given interferon associated gene in monocytes for the different mouse groups on day 2 and day 5. **(C)** Dot plot showing the normalized mRNA expression levels for interferon receptor (Ifnr) genes in B cells for the different mouse groups on days 2 and 5.

To investigate the possible reasons for the pronounced B cell differentiation impairment in the IE mice, we next focused on the increased expression of interferon (Ifn) associated genes detected in IE mice 5 days post osteotomy (**Figure 4B**). Gene module scoring revealed that monocytes and neutrophils showed a significant increase (**Supplementary Figure S5A**) in the expression of Ifn associated genes, including Irf7, Dhx58 and Parp12 detected with pseudobulk differential expression analysis in the hematoma of the IE mice from 2 to 5 days post osteotomy (**Figure 6A**). We next compared the average expression of Ifn associated genes in monocytes between different mouse groups and confirmed that the strongest increase was in IE animals 5 days post osteotomy, while none of these genes were found to be differentially expressed in NE or young animals (**Figure 6B**, **Supplementary Figures S5B, C**).

In the bone marrow (BM), B cell development is strongly influenced by interleukin 7 (IL-7) (45–49), since type I interferons produced by macrophages can inhibit IL-7-driven growth of B lineage cells and thus arrest their growth (50, 51). Therefore, the detected increased expression of Ifn associated genes such as Irf7 could indicate a positive induction of interferon levels and their secretion from monocytes, which could in turn stimulate B cells. Comparison of the expression of Ifn and Il7 receptor molecules in B cells revealed a decrease in the expression of Il7r in IE mice 5 days post osteotomy (**Figure 6C**), suggesting a possible decrease in signaling via Il7r, and consequently impaired B cell differentiation. Conversely, Ifngr1 expression was increased in B cells, which may explain an increase via Ifngr1 signaling, also supporting decreased B cell production. In summary, in IE animals, the impairment of B cell differentiation may be affected by the specific increase in Ifn associated genes in monocytes and the modified expression of their target molecules in B cells.

## Discussion

In this study, we investigated the B cell phenotype during the early inflammatory phase of bone healing at the single cell level by using a mouse model that mimics “inflamm-aged/immune-aged” individuals (10, 28). The novelty of our work lies in the delineation of B cell differentiation impairment that occurs in the hematoma during the early inflammatory phase post osteotomy, with the most pronounced block identified in IE rather than NE and young animals (**Figure 5**). In addition, these results were obtained by the systematic single cell transcriptomic analysis of BM cells isolated from female mice with differential immune experience and the subsequent bioinformatic and statistical analysis. Although we detected a relatively low number of BM resident cells in our study (~12.000), our single cell proteo-genomics based method recovered a higher number of cells than other previously published murine BM scRNA-seq datasets (27, 30, 31) (**Supplementary Figure S1E**). Nevertheless, due to our experimental setup, our findings are limited to female mice and remain to be confirmed for male animals. We decided on such an experimental setup due to the fact that female mice are known to have slower fracture healing process than male mice (75) and therefore enable a fairer characterization of the healing process, so that the bone healing outcome is not overestimated.

Earlier studies have noted the potential functional importance of B cells during bone formation and healing, since they regulate osteoclast activity (52, 53), and invade the fracture gap during the inflammatory phase and hard callus formation (26). The lack of regulatory B cells (Bregs) has been associated with delayed or non-healing fractures (PMID: 28008635 & 26303993). We were unfortunately not able to analyze Bregs, since Il10 expression, which is the hallmark of Bregs, was not detected. In future work, we plan to enrich for B cells and evaluate potentially interesting subpopulations such as Bregs. Recently, Zhang et al. applied single cell transcriptomics to a murine fracture model and found that B cells vary in number during early healing stages (27). Moreover, Molitoris et al. (54) found that the number of B cells in the callus fractures of young and aged mice gradually decreased from day 0 to day 7 after fracture, with the decrease being more pronounced in the aged mice. These findings are partially consistent with our results, since we found a general decrease of B cell differentiation in osteotomy hematoma of all mouse groups with different levels of immune experience (**Figure 5**). Importantly, our results also showed that while the B cell differentiation is generally impaired in the early phase of bone healing, this effect was much more pronounced in IE than in NE or young animals (**Figure 5**).

Although no differences in B cell proportions were observed in either NE or young mice, a significant decrease was found in the hematoma of IE mice 5 days post osteotomy (**Figure 3**). These results correlate well with the respective decrease in the pseudotime values corresponding to B cell differentiation. However, the pseudotime values in the NE mice at 2 days post injury were higher in the distal than in the hematoma and proximal tissue (**Supplementary Figure S4B**), suggesting that B cell differentiation 2 days post injury in the NE animals is inhibited in the hematoma and proximal tissue but not inhibited in the distal tissue, which also corresponds to the trend towards higher distal B cell proportion (**Supplementary Figure S2A**). Nevertheless, the detected difference in the B cell differentiation from 2 to 5 days post osteotomy in the distal tissue of NE and young mice (**Supplementary Figure S4B**) is not reflected in the decreased B cell proportion (**Figure 3C**). We speculate that other unknown factors, such as induction of unknown genes that promote differentiation or inhibit cell death, contribute to this discrepancy and could be addressed in further studies.

Hallmarks of aged adaptive immune system include systemic chronic inflammation (13–15, 55) and a decrease in the differentiation of HSCs into T and B cells (56, 57). As a result, fewer adaptive immune cell precursors are present in elderly people (13). Even though previous studies mostly focused on the age-related changes in T cells and their impact on the healing outcome (10, 11), it is widely known that B cell phenotypes are altered in many inflammatory diseases (25) and that B cells impact osteoclastogenesis (58, 59). Therefore, the highest decrease in the B cell proportion during bone healing was expected in IE mice, since those animals in fact modeled the compromised immune system found in some elderly individuals. Nevertheless, our cell proportion quantification (**Figure 3**, **Supplementary Figure S2**) only allowed us to compare relative fractions between mice groups, and we were unable to clearly delineate the reason for B cell proportion decrease in IE mice. However, based on the results of our trajectory inference analysis (**Figure 5**), lower B cell proportion in IE mice is most likely due to decreased absolute number of mature B cells because of the decreased production of mature B cells, rather than increased cell death of specific B cell subsets.

Mature B cells are responsible for the production of a large fraction of bone marrow osteoprotegerin (OPG) (60, 61), which acts as decoy for the transcription factor RANKL, thereby suppressing osteoclastogenesis and bone resorption (62, 63). In addition, mice lacking B cells in the bone marrow showed an OPG-deficient and a frequent osteoporotic bone phenotype (61). Since we found the most extensive pseudotime decrease in the IE mice, we consequently expect lower OPG secretion and extensive bone resorption, the process which unfavorably impacts the bone healing outcome (61, 64–66). Further phenotypic and mechanistic insights into B cell differentiation in the NE and IE condition are required to substantiate these findings.

In this study, we also observed an increased expression of interferon-associated genes in monocytes (**Figures 6A, B**), suggesting that a number of effectors such as type I and II interferons, as well as Il7 could play an important role in the pronounced impairment of BM B cell differentiation in IE animals. Interferon-associated genes with increased expression included Irf7 (**Figures 6A, B**), a transcription factor that controls the expression interferons, such as Ifna, Ifnb and Ifng (67, 68). In fact, we detected an increase in the Ifngr1 expression in B cells 5 days post osteotomy (**Figure 6C**), which may indicate that the inhibitory effect of Ifngr1 signaling via Ifng would be more pronounced in IE rather than NE or young animals, a result which will have to be confirmed in our future work using orthogonal methods.

Since Il7 is required for B cell development in mice (69–72) and is produced by stromal cells during early B cell lymphopoiesis (73, 74), we also analyzed the Il7r expression in B cells. We detected the lowest drop in Il7r expression from day 2 to day 5 in the IE mice (**Figure 6C**), which may explain the more pronounced impairment of B cell differentiation in these animals, since deficient Il7r signaling may lead to impaired B cell development (45). In addition, macrophages can produce type I interferons and thereby inhibit the effect of Il7 on B cell development (50). However, we cannot exclude that the detected decrease in Il7r is a consequence of decreased B cell number in IE mice 5 days post osteotomy. Since our experimental setup did not allow for the detection of stromal cells, which typically secrete Il7, further experiments have to be done to explore the possibility that they play a role in the B cell maturation impairment during bone regeneration.

In summary, our results point to a newly discovered impairment of B cell differentiation during the inflammatory phase of bone healing in IE mice and suggest that other BM cells could interact with B cells to influence their maturation. Additional work to determine the extent of such cell-cell interactions and the molecules that regulate the observed defect may prove valuable as candidate therapeutic targets for the improvement of the healing outcome in elderly bone fracture patients.

## Data Availability

Single cell RNA-seq data are available at GEO (Gene Expression Omnibus) under accession GSE273792. All analysis scripts and processed data are publicly available from GitHub at http://github.com/bihealth/fracture-healing-and-aging-scseq.
