# Supplementary figures and images for: Pronounced impairment of B cell differentiation during bone regeneration in adult immune experienced mice
Source: Front Immunol. 2025 Mar 3;16:1511902. doi: 10.3389/fimmu.2025.1511902 (PMC11911212; doi:10.3389/fimmu.2025.1511902)

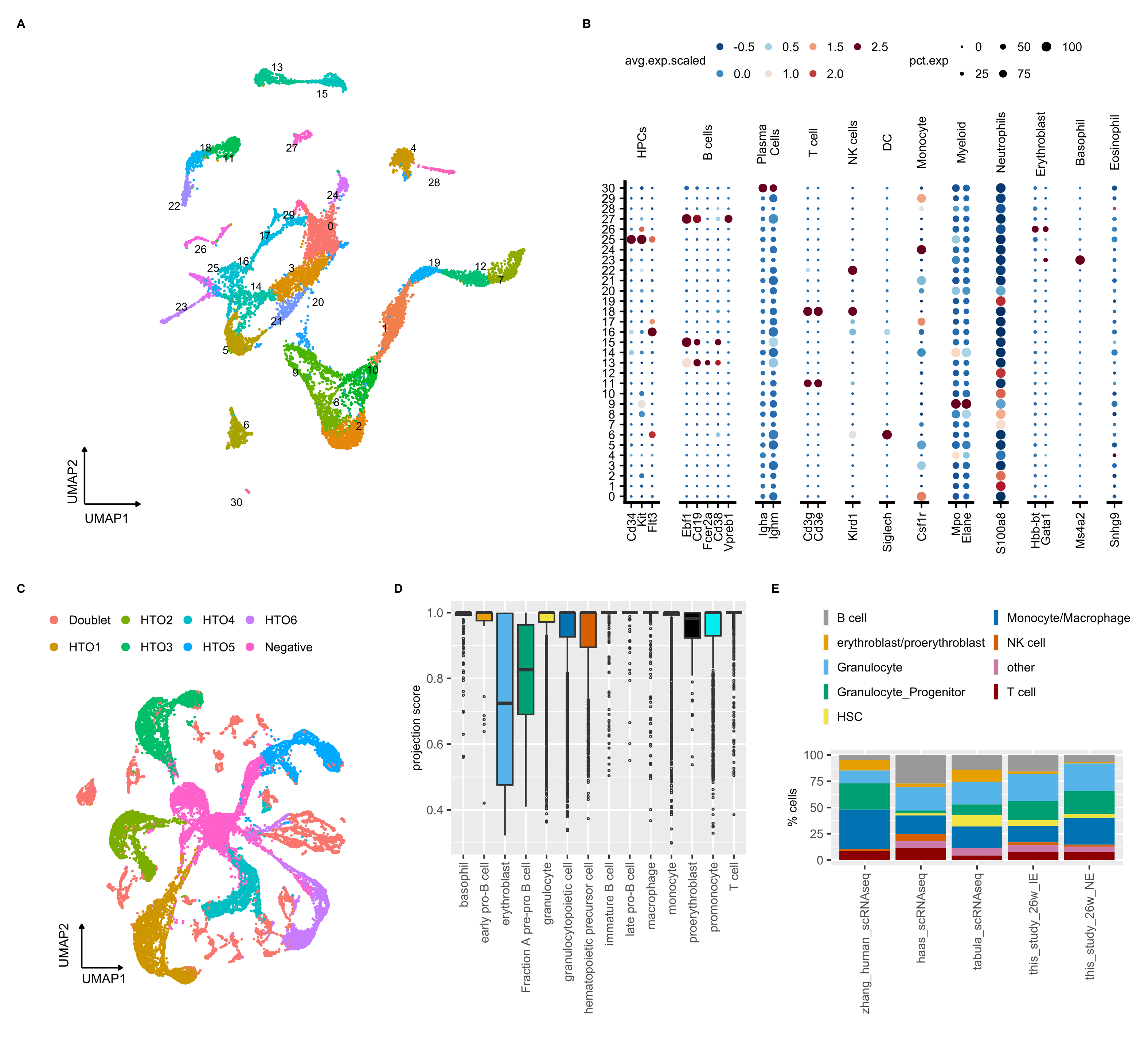

Supplement: Supplementary file 1 [file Image1.tiff]

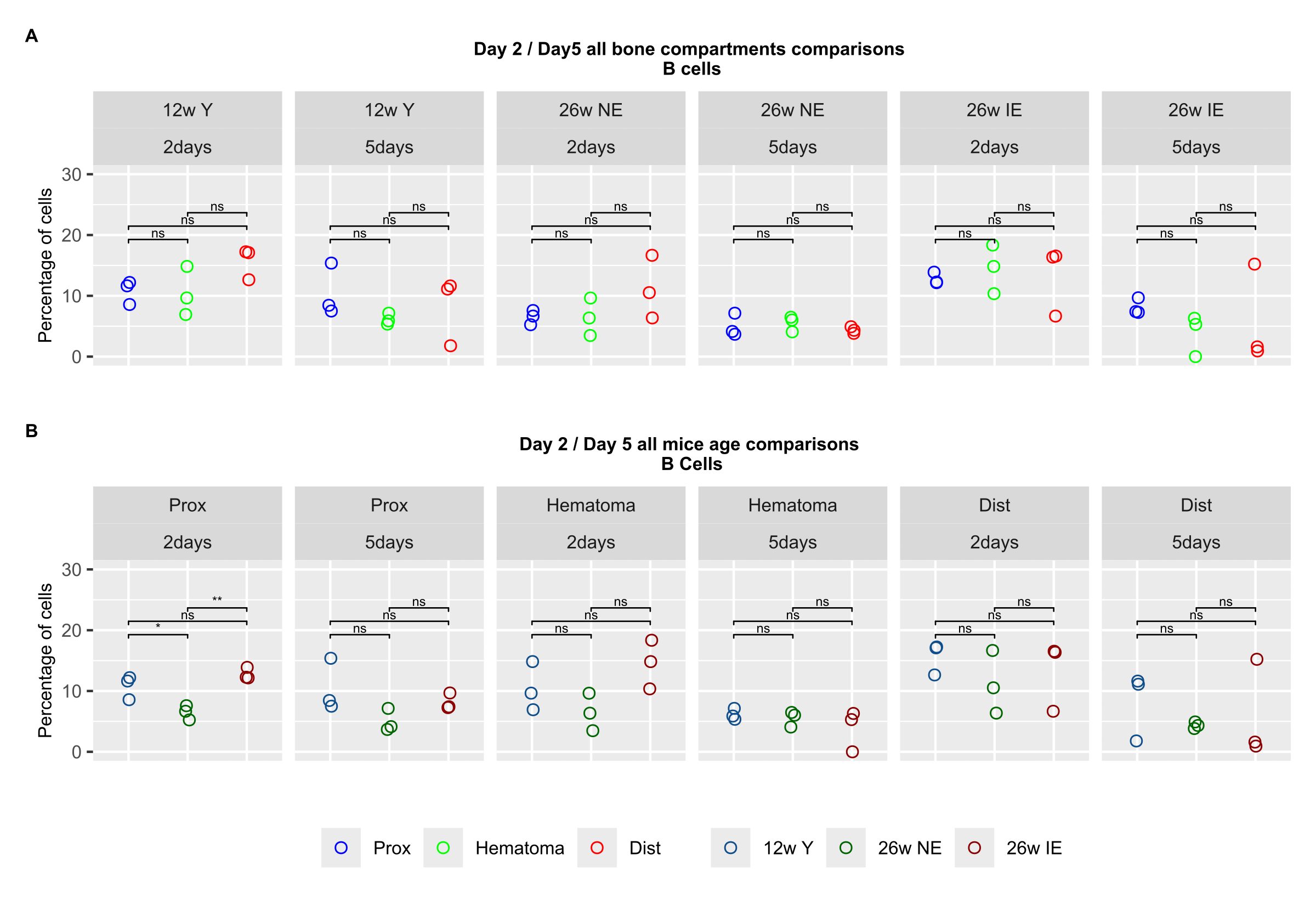

Supplement: Supplementary file 2 [file Image2.tiff]

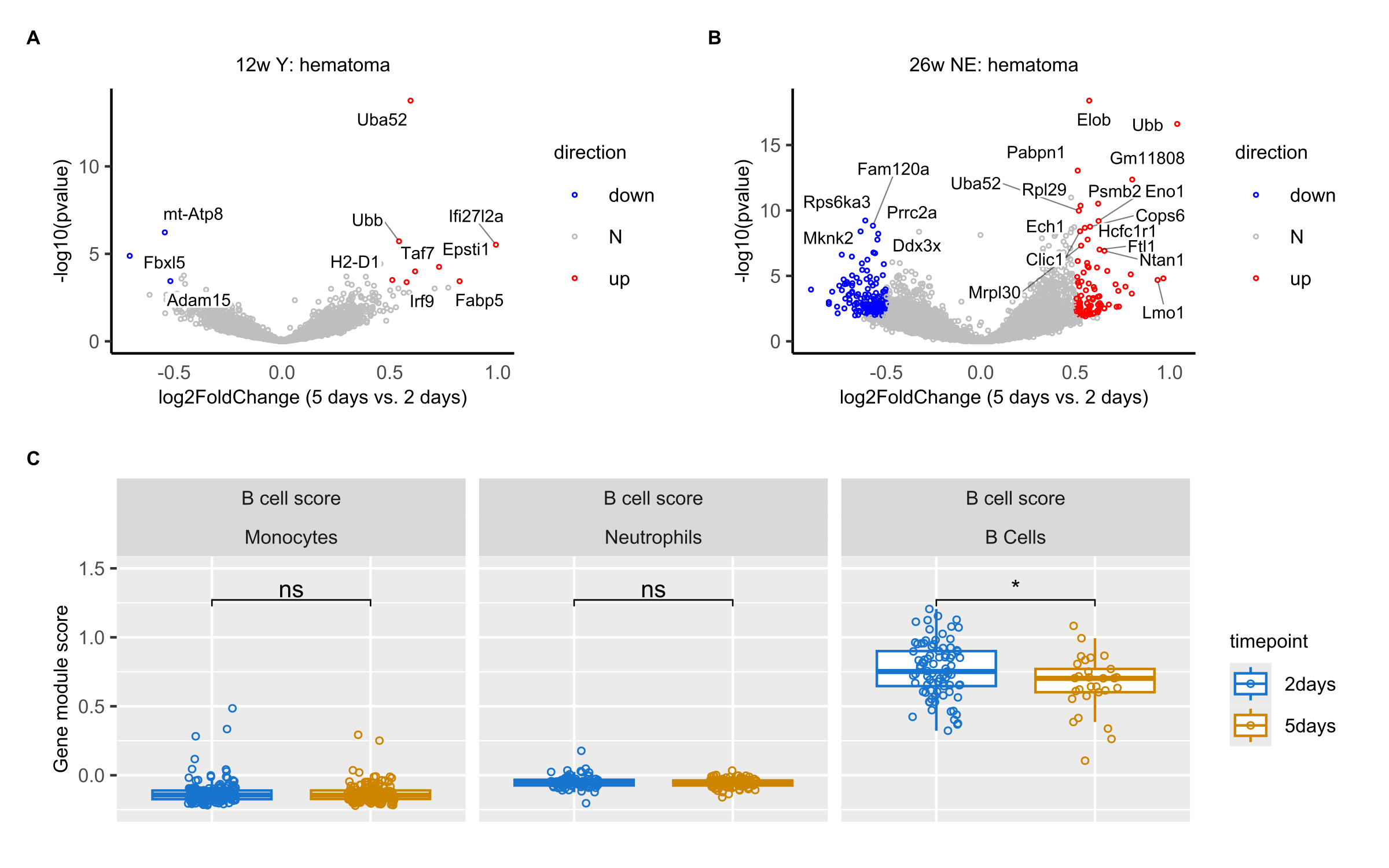

Supplement: Supplementary file 3 [file Image3.tiff]

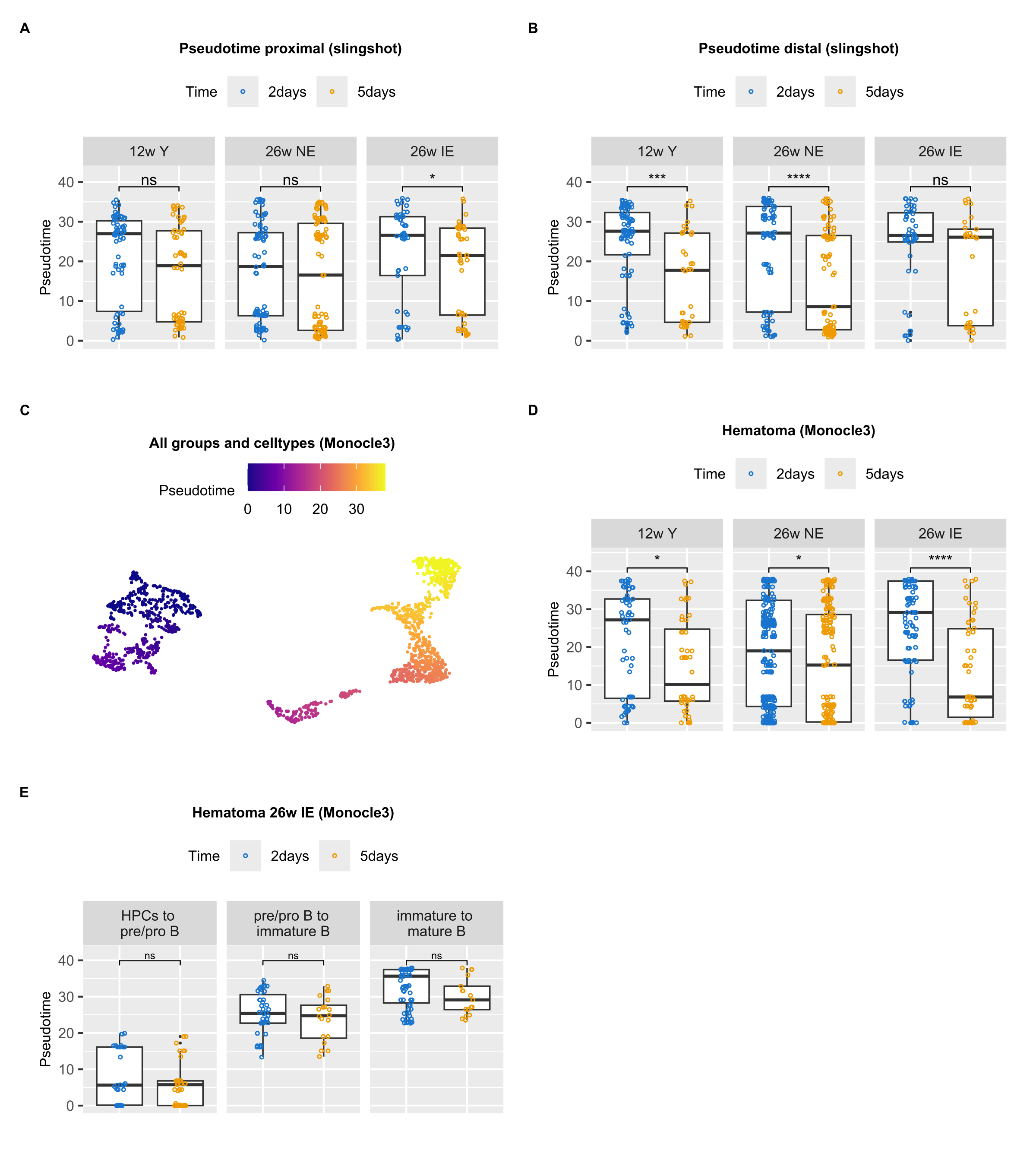

Supplement: Supplementary file 4 [file Image4.tiff]

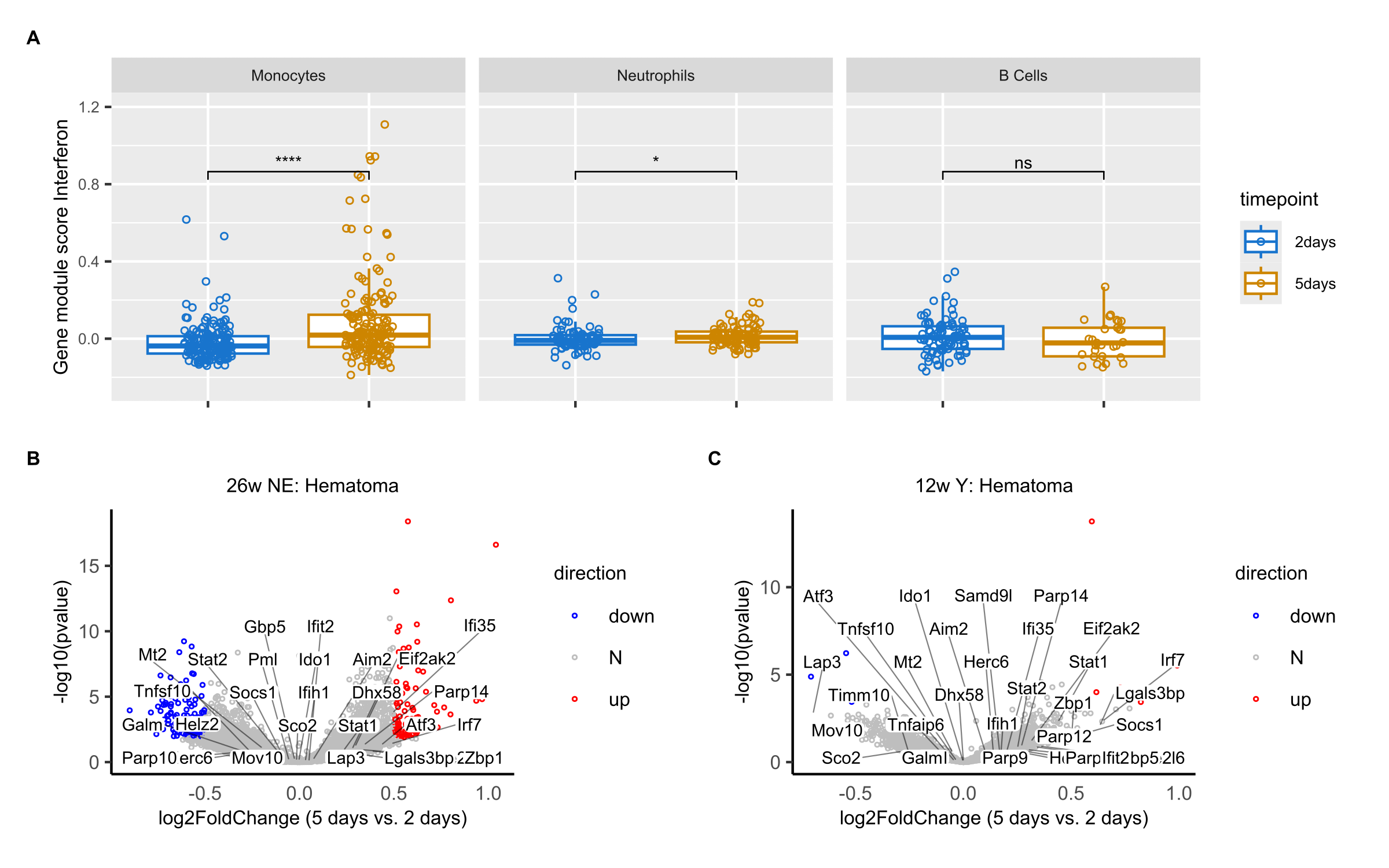

Supplement: Supplementary file 5 [file Image5.tiff]
