# Supplementary material for: Pronounced impairment of B cell differentiation during bone regeneration in adult immune experienced mice
Source: Front Immunol. 2025 Mar 3;16:1511902. doi: 10.3389/fimmu.2025.1511902 (PMC11911212; doi:10.3389/fimmu.2025.1511902)
Supplement: Supplementary file 6 [file DataSheet1.docx]

Supplementary Material

# Supplementary Data

Supplementary Material should be uploaded separately on submission. Please include any supplementary data, figures and/or tables.

Supplementary material is not typeset so please ensure that all information is clearly presented, the appropriate caption is included in the file and not in the manuscript, and that the style conforms to the rest of the article.

# Supplementary Figures and Tables

For more information on Supplementary Material and for details on the different file types accepted, please see [here](https://www.frontiersin.org/guidelines/author-guidelines#supplementary-material).

## Supplementary Figures

**
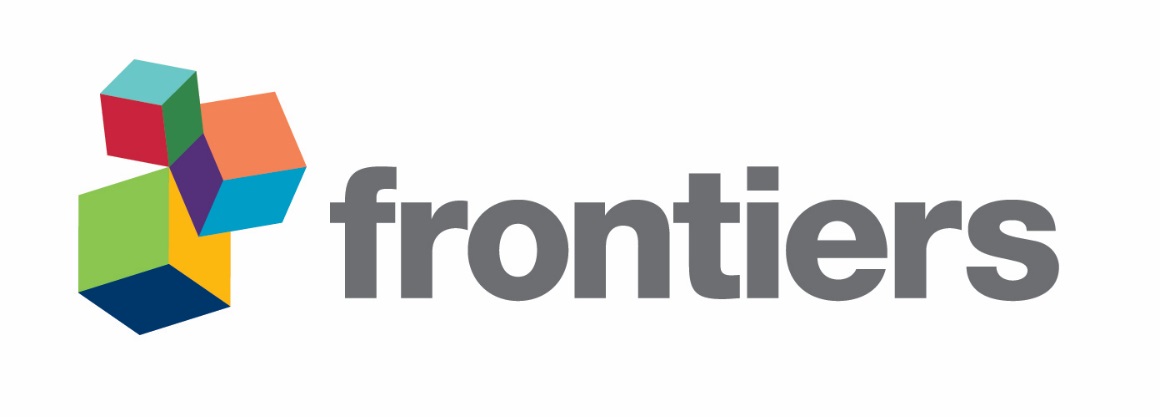
**

**Supplemental Figure 1.** Multimodal single cell analysis of mouse osteotomy bone marrow tissue detects different immune cell types. **a)** UMAP embedding of cell clusters detected in this study. Cell clusters are shown in different colors (numbers correspond to the clusters detected after Seurat standard processing). **b)** Dot plot of normalized marker gene expression values and percentage of cells per cluster that express a given marker gene. **c)** UMAP embedding showing cell-wise HTO assignments in all detected cells. Doublets and HTO-negative cells were excluded from further analysis. **d)** Estimated cell-wise scores for the projection of label transfer from Tabula Muris cell annotation. **e)** Comparative analysis of cell proportion results from murine bone marrow datasets, including this study.

**Supplemental Figure 2.** B cell proportion analysis in different bone marrow regions across all mouse groups reveals no significant differences. **a-b)** The percentage of B cells was calculated for each sample with respect to mouse age (young, NE, IE) for the different bone marrow regions on day 2 and day 5 **(a)** and with respect to the different bone marrow regions (proximal, hematoma, distal) for the different mouse groups on day 2 and day 5 **(b)** post osteotomy. Significant differences in cell proportion were evaluated using paired two-sided Student’s t-test.

**Supplemental Figure 3.** Expression of genes in the post-osteotomy hematoma of young and non-IE mice. **a-b)** Volcano plot of log2-transformed fold changes (5 days vs. 2 days) versus negative log10-transformed p-values (Wald test) in young **(a)** and NE mice **(b)**. Differentially expressed genes (adjusted p-value < 0.1 and absolute log2-transformed fold change > 0.5) are marked in red (up, increased), blue (down, decreased), or grey (N, non-differential). c) Box plots of LI.M47.0 gene module scores per cell for various cell types at 2 and 5 days post osteotomy in the hematoma of IE mice. Wilcoxon rank sum test was used for comparisons. The lower and upper hinges of box plots correspond to the 25th and 75th percentiles, respectively. Center lines of box plots depict the median values. Significant differences are indicated with asterisks (*p< 0.05)

**Supplemental Figure 4.** B cell differentiation in the osteotomy hematoma of the different mouse groups. **a-b)** Box plots of pseudotime values 2 and 5 days post osteotomy in the proximal **(a)** and distal **(b**) BM of the different mouse groups (young, NE, IE). Wilcoxon rank sum test was used for comparisons. The lower and upper hinges of box plots correspond to the 25th and 75th percentiles, respectively. Center lines of box plots depict the median values. Significant differences are indicated with asterisks (*p < 0.05, **p<0.01, ***p<0.001, ****p< 0.0001). **c)** UMAP embedding of B cells and HPCs with corresponding pseudotime values from Monocle3. **d)** Box plots of pseudotime values 2 and 5 days post osteotomy in the hematoma of different mouse groups (young, NE, IE) using Monocle3. **e)** Box plots of pseudotime values 2 and 5 days post osteotomy in the hematoma of IE mice for different cell types along the pseudotime trajectory using Monocle3. Wilcoxon rank sum test was used for comparisons.

**Supplemental Figure 5.** Differential expression of Ifn response genes. **a)** Cell-wise interferon gene set expression score in different cell types. Box plots of DC.M3.4 gene module scores per cell at 2 and 5 days post osteotomy in the hematoma of IE mice. Wilcoxon rank sum test was used for comparisons. The lower and upper hinges of box plots correspond to the 25th and 75th percentiles, respectively. Center lines of box plots depict the median values. Significant differences are indicated with asterisks (*p< 0.05, ****p<0.0001). **b-c)** Volcano plot of log2-transformed fold changes (5 days vs. 2 days) versus negative log10-transformed p-values (Wald test) in the hematoma of NE **(b)** and young mice **(c)**. Genes included in the “Interferon” gene set (DC.M3.4) are indicated along with all differentially expressed genes (adjusted p-value < 0.1 and absolute log2-transformed fold change > 0.5), and colored in red (up, increased), blue (down, decreased), or grey (N, non-differential).

## Supplementary Tables

**Supplemental Table 1.** Overview of the experimental groups on days 2 and 5 post osteotomy.

**Supplemental Table 2.** Cell proportion values per mouse on days 2 and 5 post osteotomy.
